# Review of Diet Quality Indices that can be Applied to the Environmental Assessment of Foods and Diets

**DOI:** 10.1007/s13668-024-00540-0

**Published:** 2024-04-16

**Authors:** Alba Reguant-Closa, Dario Pedolin, Moritz Herrmann, Thomas Nemecek

**Affiliations:** Life Cycle Assessment Research Group, 191, CH-8046 Reckenholzstrasse, Zurich Switzerland

**Keywords:** Sustainability, Dietary indices, Life cycle assessment, Diet quality, Healthy diets, Nutrient profile

## Abstract

**Purpose of Review:**

The aim was to identify indices of diet quality and health that could be applied to the environmental assessment of foods in order to provide metrics that collectively assess nutritional, health and environmental dimensions.

**Recent Findings:**

The review identified five major groups of indices: nutrient-food quantity-based; guideline-based; diversity-based; nutrient quality-based; health-based. Nutrient-food quantity-based and guideline type indices were the most frequently used to evaluate diet quality. Scaled assessment using a nutritional functional unit is the most common integration of diet quality with the environmental analysis of foods. There are fewer indices that measure the heath impacts of foods, but epidemiological dietary risk factors seem a promising approach to integrate diet and health impacts into the environmental assessment of foods.

**Summary:**

Five groups of nutritional and health indices were identified that can be applied when performing an environmental assessment of foods. This review proposes different methodological insights when doing such assessments to ensure transparency and comparability of the results.

**Supplementary Information:**

The online version contains supplementary material available at 10.1007/s13668-024-00540-0.

## Introduction

Ensuring high quality and sufficient quantity of food to feed the growing world population has been a concern for the past century. Nevertheless, doing so in a sustainable manner guaranteeing high diet quality has become one of the greatest challenges for the XXI century [1•, 2•, 3, 4]. Finding indicators to evaluate aspects of diet quality, health and environmental impact of foods has become of utmost interest in the last years to ensure that a more environmentally friendly diet is also a healthy and nutritious one.

Ensuring adequate diet quality is relevant for both diets that are inadequate in energy content but also in nutrient content. Indeed, the worldwide increase in obesity, overweight and non-communicable diseases, especially in developed countries, is partly associated with a change in dietary habits [5]. These new dietary trends are typically characterised by a lower consumption of fresh and naturally high fibre products (i.e., vegetables, fruits, pulses, whole grains) and an increased consumption of sweet beverages and processed foods high in calories, sugars, salt and fats. Therefore, many new initiatives address the obesity epidemic to promote a more nutritious diet but also to prompt the food industry to produce nutritious food products and beverages [6]. With that purpose, different nutritional indices have been developed with the aim of classifying diets, meals and foods with regard to diet quality. When discussing diet quality, often only nutrient composition is considered which is known as the reductionist approach [7]. However, meals and diets contain a wide variety of foods, composed of different nutrients that interact to enhance or limit nutrient bioavailability and can often affect the final nutritional quality of the meal. While it is challenging to develop a sole or composite indicator able to incorporate all the different aspects of diet quality, a multidimensional approach and a clear definition of its components are important [8, 9].

Many indicators of diet quality have been shown to be associated with health outcomes but there are some challenges with this strategy. While it is generally true that a good diet quality will have a positive effect on health and that some nutrients and food groups have been associated with positive (i.e., whole grains) or negative (i.e., red or processed meat) health outcomes, it is not a sine qua non-relationship as there are many factors that affect health [10]. Some studies include a health assessment of the diets including aspects of mortality/morbidity [11, 12]. However, those studies are generally focussed on one aspect of health/illness such as prevalence of specific diseases (i.e., diabetes) and do not evaluate the wider health impacts of foods [13, 14]. It is noteworthy that, the World Health Organization (WHO) definition of health emphasises the multidimensional facets including the physical, mental and social dimensions, again, suggesting that a reductionist approach to health should be avoided.

Recently, there has been more interest to link dietary patterns with not only human health but also planetary health [1, 15]. The increasing concerns about climate change have also raised awareness about including environmental principles in dietary recommendations [16]. This strengthens the need to evaluate food not only from a nutritional, health and/or environmental perspective, but to find quantitative indicators and frameworks that integrate nutrition, health as well as environmental dimensions into the evaluation of foods [17]. In addition, ability to quantify small changes, which could have a large impact on all three dimensions (nutrition, health and environment), may help populations to make realistic shifts towards consuming a more sustainable diet [18, 19••].

**Fig. 1:**
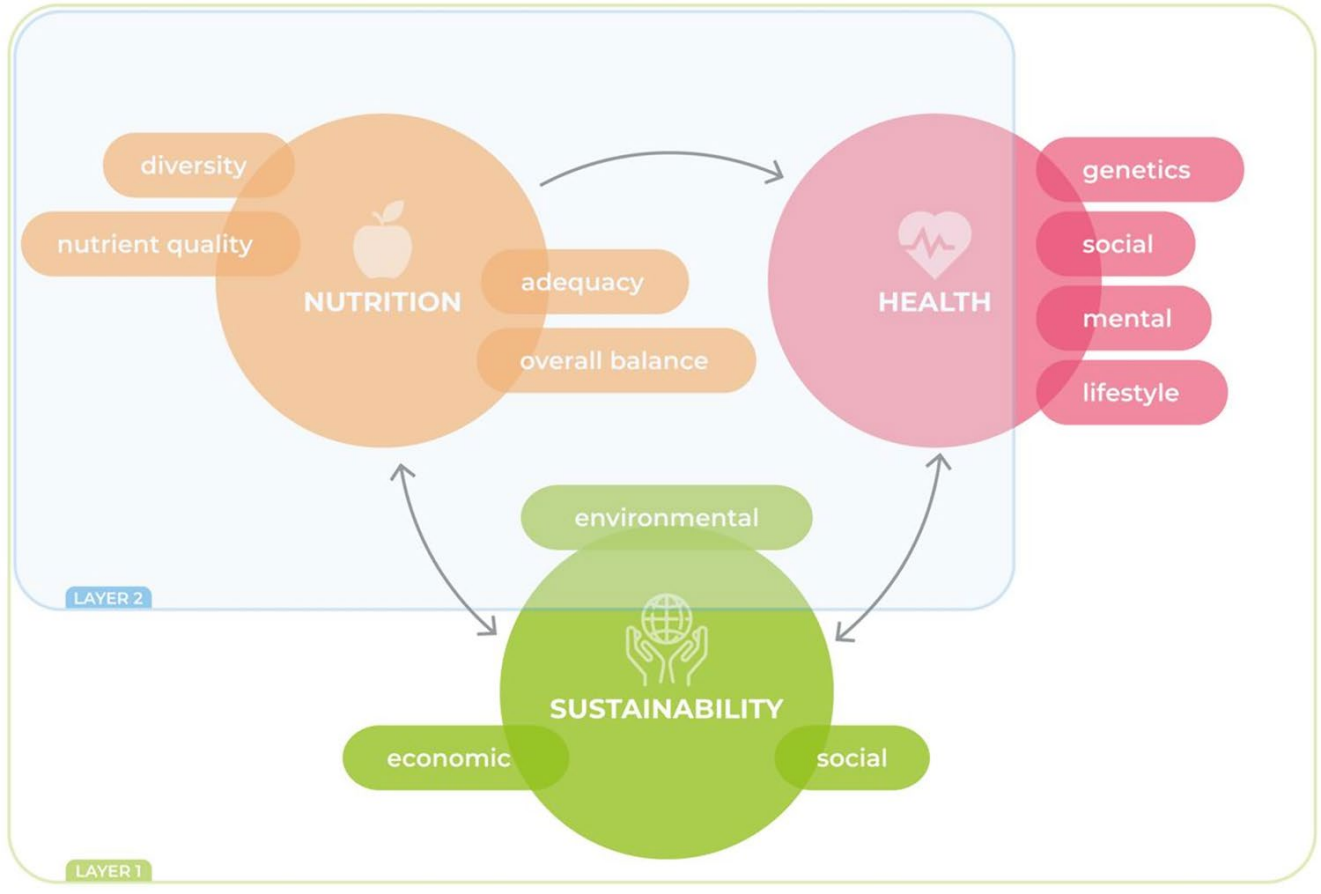
Definition of the scope of the study. Layer 1 represents the whole nutritional, health and sustainability dimensions. Layer 2 represents the inclusion of nutrition, dietary impacts on health and environmental dimensions of food which is the focus of this study

Though this is a vast and growing research area, this paper aims to (1) review the potential of existing metrics of diet quality and/or health to be applied in the context of environmental sustainability analysis of foods, meals and diets; (2) discuss strengths and limitations of the different metrics; and (3) recommend possible applications and future directions of research. As diet quality, health and sustainability are each comprised of multidimensional concepts; Fig. 1 defines the scope of the literature review for this study.

## Literature Review

### Methodology

**Fig. 2:**
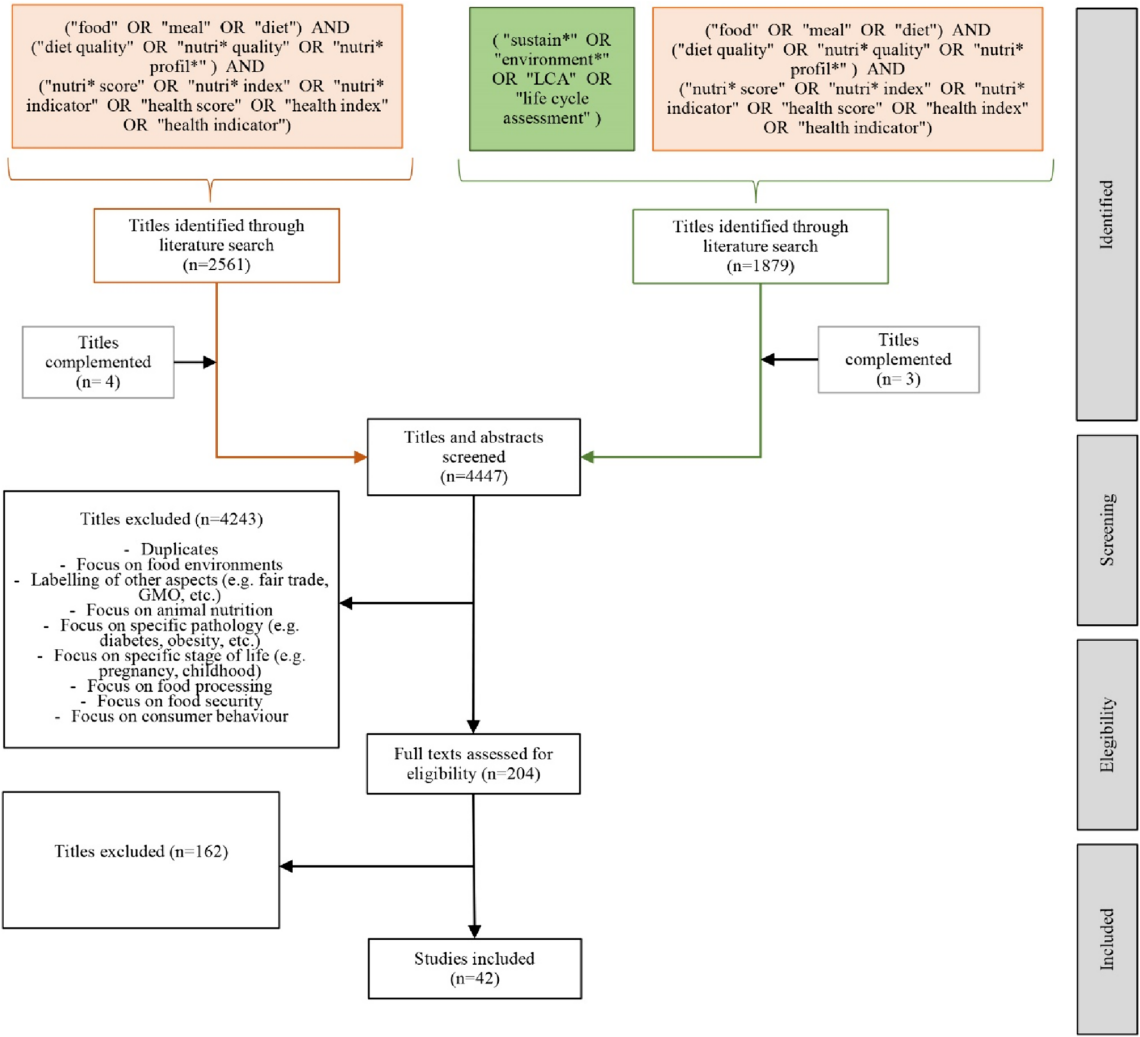
Literature search method protocol. Exclusion criteria are defined

The literature search was conducted in Scopus during March 2023 following the PRISMA statement protocol [20]. The review was structured in two search strategies to capture (a) diet quality metrics and (b) their application for the environmental assessment of foods (see Fig. 2). The first search strategies focused on diet quality and only review papers of dietary metrics or methodological studies developing or validating dietary indices were included. All dietary indices that complied with the following were included: (1) nutrient/food based; (2) measure one or more dimensions of diet quality or health. As described by [8], diet quality is multidimensional and also extends notions of food safety, cultural acceptance and satiation but these were not included in the scope of this study. The second search strategy included the same keywords plus the environmental terms and included only review or methodological papers assessing and or discussing the framework for nutritional health and environment (NHE) assessment of foods. The reason for this was to ensure that broader diet quality metrics were included in the review, as potentially relevant for NHE assessment of foods even if they are not used yet on the current literature in combination with environmental assessment in the current literature. The literature review was limited to English language from January 2000 until February 2023. In addition, grey literature (i.e., FAO reports) and following up references in relevant studies from the initial search (snowball review) were also included. Selected papers were screened and excluded based on inclusion and exclusion criteria for the abstract, keywords, title and then by the full body of the paper. Finally, results from the literature review were analysed to identify (1) descriptions, development or validation of diet quality or health metrics; (2) methodological considerations; and (3) potential and limitations of diet quality or health metrics for the assessment of NHE dimensions of foods. From the 4447 articles on the initial literature search, 42 where finally considered in this review (see Fig. 2).

## Results

Diet quality is poorly defined in most studies and more information is needed to specify which dimension of diet quality is assessed (e.g., nutrient content, diversity, etc.) [9, 21]. Additionally, some studies use the term indicator, others use index and other use score [21, 22]. This inconsistency in the literature adds confusion to the topic. As there is no standardized and well-defined framework of diet quality or the terms used to measure it, we agreed to the following definition for this paper. Diet quality refers to the aspects of diet adequacy, diet variety/diversity, moderation and overall balance. In addition, its definition will vary depending on the target population’s specific needs (age, gender, physical activity) and cultural and social context and consequently dependent to a specific set of dietary recommendations/guidelines. For this paper, index is defined as the result from a mathematical calculation, which typically includes several nutrients and/or food groups. Nutritional indices evaluate, at least partly, the nutritional quality of food items. On the other side, indicator is a value that points to the status of what is being measured and can be used to predict or take action. Indicators can be single or composite (they summarize information). For this paper, dietary and health indices are considered as the result of mathematical algorithms that measure characteristics of diet quality (i.e., the healthy eating index). Each index will give a value, which is an indication of a specific status of the aspect of diet quality that is measuring but cannot be considered alone as a direct relationship of overall diet quality.

## Review of Dietary and Health Indices

For the purpose of this review, the indices identified during the literature review assessing diet quality were divided in five different groups (see Table 1). Table S1 provides a list of the included studies on the review classified by index category.

**Table 1:** Group classification of dietary and health indices

Group classification		Characteristics	Examples
**Group A:** Nutrient/food quantity based indices	A1	Ratio between nutrient/ food content and reference amount (i.e., DRI) for qualifying and disqualifying nutrients and/or foods	• Nutrient rich food indices (NRF) • Nutrient balance concept (NBC)
	A2	Categorized or dichotomous indices used as front-of-package nutritional labels (FOPNL)	• Nutri-Score (NS) • Health star rating system
**Group B:** Guideline based indices		Based on the adherence to specific guidelines on healthy eating	• Healthy eating index (HEI) • Mediterranean eating index (MEI)
**Group C:** Diversity based indices		Based on nutrients/food group diversity	• Dietary diversity score (DDS)
**Group D:** Nutrient quality-based indices		Considers nutrient quality characteristics specific to one or more nutrients (bioavailability, digestibility, etc.)	• Digestible indispensable amino acid score (DIAAS)
**Group E:** Health based indices		It accounts for health impacts of foods and diets based on dietary risk factors	• Health nutritional index (HENI)

### Group A: Nutrient-Food Quantity-Based Indices

This group comprises indices that describe adequacy, based on the concept of nutrient profiling, as a ratio between nutrient or food content and a reference amount for qualifying (to encourage) and disqualifying (to limit) nutrients or foods [23••]. The concept of these indices is that some nutrients or foods have a positive or negative impact on diet quality. This group was divided into two sub-groups depending on their output: (A1) continuous index; (A2) categorized or dichotomous index used as front-of-package nutritional label (FOPNL). From the literature review, twenty-six studies included group A indices. Twenty-three studies included group A1 indices [17, 22, 23••, 24••, 25–28, 29••, 30–40, 41••, 42•, 43, 44], three studies included group A2 indices [42•, 45, 46] and one study discussed A1 and A2 type indices [23••]. The literature review identified the Nutrient Rich Food (NRF) family indices and variations of it as the most used A1 type index [17, 22, 23••, 24••, 25–28, 29••, 30–40, 41••]. One review assessed types of A2 indices by countries or regions, and the capacity of those to discern food characteristics and rank them [42•]. The main FOPNLs identified were the traffic lights system for the UK, the Keyhole in the Nordic European countries and Nutri-Score for several European countries that have adopted it or are in a process to review and assess its inclusion [42•, 45, 46]. However, the review stated that there is no consensus yet on which is the best FOPNL which is dependent on the nutrients considered, the type of product and the health policy priorities of each country [42•]. Type A indices can be used to evaluate foods, meals and diets but the literature review identified five main methodological adaptations to consider.

- *Reference amount:* Group A1 indices allow reference values to be adjusted to different target population, while Group A2 indices do not allow for flexibility on the cut-off points which are predefined and generally are median cut-offs [42•]. Qualifying nutrients are expressed relative to Daily Reference Intakes (DRI) and disqualifying nutrients are expressed relative to Maximal Reference Values (MRV) of the target population resultant from diet-health relationships [34, 39, 47]. When these indices include foods, those are expressed relative to their recommended intake, generally based on dietary guidelines [28].
- *Scale and results output:* For the majority, higher numbers represent higher nutritional quality and lower numbers are indicative of poorer nutritional quality. However, for the Nutri-Score, the scaling is inversed and lower numbers are indicative of better food quality [46]. In A2 type indices, the nutrient profiling algorithm is translated into a set score point system and results are shown in a visual way (i.e., graphic scale) easier to understand by consumers and aiming to help them make healthier-nutritious food choices [42•].
- *Capping:* Some indices consider capping nutrient scores at 100% of their DRI to ensure that foods with larger qualifying nutrient values do not receive a higher value [34, 39]. When evaluating single foods, capping is generally not recommended as single foods might not contain a full range of necessary nutrients [41••]. On the other hand, capping is generally recommended when assessing diets as the overall diet should be balanced.
- *Weighting/standardization:* Weighting can be applied on the nutrient/food level and is recommended when there is the need to give a higher importance to a specific nutrient or food (i.e., due to deficiencies in the population) [41••]. Regarding the energy level, energy standardization can be used, to make foods more comparable, especially when comparing nutrient densities of food products [23••, 34, 39]. The Nutrient Balance Concept (NBC) includes an energy weighting system to balance out differences between high and low energy dense foods and includes a nutrient balance indicator (NB) aiming to account for how much a food, meal or diet satisfy daily dietary requirements of qualifying nutrients [34].
- *Selection of nutrients/foods:* The literature review documented an extensive selection of Group A type indices depending on the nutrients included in the equation. The most commonly used one is the Nutrient Rich Food 9.3 (NRF9.3) as it shows better correspondence against the Healthy Eating Index (HEI) [39] but there have been developments of NRF up to 25 positive nutrients [23••]. The main rationale for inclusion or exclusion of nutrients in the index relate to the overall goal of the study and data availability [23••, 41••]. First, the goal of the study will determine whether the index needs to include a comprehensive range of nutrients or whether there is interest in specific foods or nutrients [17, 23••, 31, 39, 41••]. Second, some indices include a large list of nutrients which theoretically allows for a comprehensive generalized assessment of nutrient quality, but can only be applied if data are available in nutritional composition databases. In the case of missing data, the use of a proxy or a modification of the index will be required. Additionally, when applying these indices for composite food products with multiple ingredients, it is difficult to know the quantity of each ingredient that is included in the formulation. This is specially complicated for processed industrialized food, where the exact composition of a food is not declared. To have a more comprehensive nutrient profiling of foods, the inclusion of not only nutrients but also food groups in the NRF family indices has been discussed recently [28]. The rationale for its inclusion is that when considering only nutrients (i.e., fibre of whole grains), other components which might have a positive nutritional impact are being dismissed (i.e., antioxidants). With this aim, Drewnowski and Fulgoni [28] developed a new NRF incorporating nutrients and foods showing better alignment when validated against the HEI than when only nutrients were considered.

### Group B: Guidelines Based Indices

This group of indices evaluates the adherence to specific guidelines of healthy eating and assume that recommended guidelines improve diet quality and are healthy or they provide benefits for health (i.e., Mediterranean diet pattern). Often, these indices are well correlated with mortality rates and specific diseases such as cancer, diabetes or cardiovascular diseases. The Healthy Eating Index (HEI) and the Alternative Healthy Eating Index (AHEI) have shown good associations with the risk of major chronic diseases [48, 49]. Thus, guidelines-based indices, and especially the HEI and AHEI have become quality standards when evaluating diet quality and often used to validate group A type indices [39]. Generally, these indices evaluate nutrients and food groups from diets, but others also incorporate healthy lifestyle aspects (i.e., nap/siesta or social life) [33, 50]. The scoring system of these indices is based on a continuous point-based scale that can vary for each index but which generally adds positive points when the recommendations are followed and negative points when they are not. The majority of these indices are country (i.e., adherence to the US guidelines) or region specific (i.e., adherence to Mediterranean diet). However, a comprehensive assessment of country specific guidelines-based indices is not an aim of this review. The literature review found nineteen studies referring to indices in this category [9, 21, 22, 28, 29••, 33, 37, 39, 40, 43, 47–49, 51–56]. The most used indices are the Healthy Eating Index (HEI) and the Mediterranean Diet Score (MDS) [22, 33, 54, 55] or derivations of those two.

### Group C: Diversity Based Indices

This group of indices quantifies diversity aspects of the diet between and among food groups. These indices are based on the assumption that a higher diversity of the diet (from food groups and/or nutrients) increases diet adequacy and consequently dietary health. For example, the principle is not to include only one cereal in the diet but a wide diversity of cereals that will enrich the nutrient density of the diet. Eleven studies were found in the literature review that include diet diversity indices [9, 17, 29••, 33, 43, 47, 51, 53, 54, 56, 57]. Verger et al. [57] reviewed dietary diversity indices finding that the majority were associated with nutrient adequacy, especially micronutrients, but lacked evidence on its relationship with nutrients to limit and health outcomes. Thus, it is recommended to use a diversity index in combination with another dietary index. Also, diversity based indices are generally more relevant in rural areas of developing countries where diet diversity may be more limited [40].

### Group D: Nutrient Quality-Based Indices

Group D includes indices that consider specific nutrient quality characteristics such as bioavailability, digestibility or absorption. This is highly relevant, as nutrient quality is dependent on food type and is influenced by enhancing or limiting factors of the food matrix. The complex nature of these measurements was reflected by the lower number of studies that included this type of index. Five studies identified in the literature review included quality-based indices [17, 33, 41••, 58•, 59]. Protein quality has been extensively discussed because it plays an essential structural and functional role and is vital to support human health. In addition, plant-based proteins have in general a lower quality and a different amino acid profile than animal-based proteins. Gil et al. [33] reviewed, from a historical perspective, different methods to measure amino acid composition and protein quality of foods and concluded that the Digestible Amino Acid Score (DIAAS) should be used when possible. DIAAS is recognized as the best method to evaluate protein quality, but has its limitations as it only accounts for the limiting amino acid and currently there are insufficient accumulated digestibility data from human foods databases. Carbohydrate quality has also recently become a focus as simple carbohydrate dietary intake has increased. In fact, whole grains have been a centre of attention for their high fibre content (positive health outcomes) and low sugar content (negative health outcomes). Thus, some studies are claiming that carbohydrate quality is as an important nutritional feature for overall diet quality [33]. In addition, micronutrient quality should also be considered as the bioavailability of many micronutrients is highly dependent on the food matrix. For example, iron bioavailability is enhanced by the presence of vitamin C or limited by coffee consumption. Thus, being able to account for nutrient quality and not only quantity is highly relevant but not always easy to apply.

### Group E: Health-Based Indices

Group A to D indices are food and nutrient-based methods that assess different dimensions of diet quality. But how diet affects overall health status is also an important parameter to be considered when evaluating diets, but it is difficult to measure as many aspects influence the health status of populations (e.g., genetics, physical activity). Many nutritional indices identified in the literature review include a correlation between their results and health outcomes such as mortality or prevalence of a specific disease [21, 22, 33, 45, 46, 48, 49, 60]. Thus, diet quality scores are useful tools to test if dietary recommendations have a measurable protective effect against diseases. Eight studies identified in the review discussed health-based indices [17, 19••, 24••, 26, 29••, 37, 41••, 61••]. The newly developed Health Nutritional Index (HENI) seem a promising method to evaluate dietary health impacts [19••]. The HENI uses 15 dietary risk factors based on epidemiological data from the global burden of disease study (GBD) [10]. The HENI is expressed as micro-disability adjusted life years (µDALYs) and positive values indicate minutes of healthy life gained per reference amount of food consumed considering all 15 dietary risk factors, while negative values indicate minutes lost [19••].

## Application of Dietary and Health Indices on the NHE Assessment of Foods

Table S1 in bold displays all the studies that evaluated the environmental dimension of foods in combination with the nutritional and/or health dimension. While, nutrition and health should be incorporated when assessing the environmental impacts of foods, its integrated analysis requires methodological considerations. As defined by Hallström et al. [29••] and Guo et al. [61••], the analysis can be performed (1) parallel; (2) scaled; or (3) integrated. In a parallel assessment, a separate analysis of the nutritional, health and environmental dimensions is performed and a correlation between them is included. This type of analysis is easier to be applied, but trade-offs and synergies of each dimension are more difficult to be captured. A scaled analysis will include a separate analysis of each dimension but translated into a same scale or unit, generally a nutritional dependent functional unit (e.g., dividing the environmental impact by a dietary index). In this case, the results are generally evaluated considering the nutritional functional unit (FU) against another typical life cycle assessment functional unit (e.g., mass based unit). On the other hand, a more integrative approach will result in a sole final index which combines NHE dimensions. The integrated approach is more complex and requires a new score to be developed which considers all dimensions. In this case, methodological aspects will have to be well described and argued (e.g., weighting of each dimension) and will require further validation. Green et al. [17] discuss the pros and cons of composite indicators when evaluating the NHE of foods concluding that while these types of indices facilitate the communication to the final user who might not be expert on the topic (e.g., farmer, policy-maker, consumer), methodological and interpretation bias have to be considered cautiously. Additionally, nutritional and health dimensions could also be integrated as impacts on a life cycle assessment, but also, methodological aspects need to be considered [17, 24••, 26]. Currently, there is no consensus on which is the best option to perform a NHE analysis of foods, but the papers identified in this review agree that a common evaluation is important to properly identify synergies and trade-offs to develop recommendations of food production and consumption [29••, 41••].

The incorporation of health impacts into life cycle assessment has been addressed in a recently published review [61••]. Guo et al. [61••] reviewed methods to integrate health metrics into the environmental assessment of foods and diets. They identified eight types of health metrics where death (avoided, averted, delayed or preventable) and disability or quality adjusted life years were the most common ones used. From the literature review, three studies describe the methodological approach of the recently developed HENI index, based on the global burden of disease study (GBD) epidemiological data [19••, 24••, 26], while others discuss possible methodological applications to incorporate disability adjusted life years (DALYs) or dietary risk factors as part of the health impact category when performing an LCA [17, 24••, 41••]. In fact, DALYs can be directly added at the endpoint level on the human health category as it has the same units than other impacts considered in this category. This methodological approach is suggested by Jolliet [24••] as a possible integration to account for the health impacts of food consumption, which is typically not included in a LCA design [19••, 24••].

The dietary dimension is often included in a LCA as a nutritional-based functional unit (scaled integration), which represent the environmental results based on the nutritional impacts, also known as nutritional LCA (nLCA). McLaren et al. [41••] summarizes FUs used in nLCA and their benefits and limitations. The diet quality indicators most commonly used as FU are the nutrient quantity-based indices identified as group A in this study [27, 29••, 41••]. The methodological considerations when applying these type of indices (i.e., capping, weighting) will influence the LCA results interpretation, thus studies should be transparent on which methodology has been applied in each case with clear justification [23••, 31]. However, foods have multiple functions [24••] which are difficult to capture if only nutrient content is considered. Thus, nutrient quality indices are also used as FU, especially when evaluating specific nutrients (i.e., protein quality) [29••, 58•, 59]. From the identified studies, diversity indices are used less [17, 57]. Nevertheless, as diversity on the plate would affect diversity in the field (mirroring field to plate), it might be reasonable to assume that diversity indicators could be used in parallel to the biodiversity assessment when evaluating environmental impacts of diets. Green et al. [17] propose a classification of nutritional diversity metrics and its relationship to human health and environmental sustainability but also state that diversity metrics are rarely included in NHE assessments. Aldaya et al. [53] also discuss the importance of linking nutritional and environmental biodiversity. Waijers et al. [22] recommend in their review to include a specific diversity indicator as a complement to another dietary index (i.e., DQI-I). However, to our knowledge, no study has addressed this yet. The importance of including adherence to dietary guidelines (i.e., Group B indices in this review) in the environmental assessment of foods has been suggested [29••, 53] but our literature review did not identify any studies that have done this to date. Dietary guidelines promote a healthy eating style. Thus, type B indices could also be incorporated to assess the nutritional and health impacts of diets when performing an LCA.

A more integrative approach of nutrition has also been identified in the reviewed studies. Strid et al. [25] suggested two methods to evaluate the environmental and nutrition dimensions of different foods. They analysed products using an integrated approach (using a combination of nutritional and environmental values) and a parallel approach (using an indicator for the environmental (climate change) and nutritional (nutrient density) dimension). The authors concluded that the integrated indicator had a better coherence with the recommended dietary intakes. In addition, the results indicated positive synergies between climate impact and nutrient density for a good percentage of foods, especially for those recommended in the dietary guidelines [25]. Jolliet [24••] proposes a framework to integrate nutritional impacts associated to the consumption of foods in Life Cycle Assessment. Hallström et al. [29••] identified in their review four studies that included an integrated sustainability score based on environmental and nutritional impacts but a more comprehensive analysis of combining both dimensions is still required. While a more integrative approach or common score is desirable for an easier assessment of sustainable foods, there are still several methodological aspects (e.g., how many impact categories or diet quality aspects can be included in an integrated index) that need to be considered and so, more research is needed in this area to arrive to determining conclusions. Aiming to facilitate the integration of the NHE dimensions of foods, meals and diets, Table 2 provides a summary of the findings of this literature review and possible applications.

**Table 2:** Diet quality and health index checking list for the NHE assessment of foods.

Aim	Type of index	Most used	Additional information	How to include in LCA
Nutrient/food quantity	Nutrient profiling	Nutrient rich Index (NRF)	Adapt the DRI and MRV to the target population Include/exclude or weight nutrients depending on deficiencies Include graphical version for consumer (only in A2 indices) Include food groups and not only nutrients	To use as functional unit (all or only qualifying nutrients). Parallel assessment
Diet adequacy	Guidelines based indices	Healthy Eating index (HEI)	Adapt to country or population specific guidelines Include other aspects of lifestyle if possible	Parallel assessment
Diet diversity	Diversity based indices	Diet Diversity Score (DDS)	If possible, consider in and within food groups	Include in the biodiversity impact category? Parallel to biodiversity assessment
Nutrient quality	Nutrient quality indices	Depending on which nutrients are considered	If possible, consider interaction within the food matrix When data is available use in vitro bioavailability data	To use as functional unit Parallel assessment
Health	Consider the influence of dietary risk factors into health outcomes	Health Nutritional Index (HENI)	Adapt dietary risk factors to target country and population	Include in the health impact category Parallel assessment

## Discussion

The literature review identified a broad range of dietary and health indices that are currently used to measure different aspects of diet quality or health effects of foods, meals or diets. Therefore, it is crucial to define which aspect of diet quality is being evaluated and accordingly choose the appropriate nutritional index. A large proportion of the reviewed studies focused on nutrient profile type of metrics or adherence to specific guidelines. To facilitate the integration of specific characteristics of diet quality and health when evaluating foods, meals and diets, this study proposes a new classification of different indices from A to E described in Table 1. Not all indices identified in the dietary-health quality literature review were used in the NHE studies evaluated. When considering NHE assessment of foods, nutrient profiling indices were the most used, generally as FU in a nLCA study. However, different papers discuss the need for a more integrated approach. Thus, there is a potential for a more holistic integration of diet quality and health when evaluating its environmental impact.

Data availability is a limiting factor when assessing diet quality of foods. First, nutritional composition databases contain a limited number of nutrients. Thus, nutrient profiling might not account for some nutrients that are relevant for the target population (i.e., in case of deficiencies) [23••, 62]. In some cases, databases do not differentiate between added or naturally contained nutrients which can make a difference when applying dietary indices (e.g., sugar naturally contained in fruits vs added sugar). Second, indices that focus on nutrient quality require specific data on the bioavailability of nutrients that are not often available (e.g., amino acid digestibility). In addition, the food matrix of the analysed meal or diet will influence the bioavailability of specific nutrients (enhancing or limiting it) and to our knowledge, there is no comprehensive database that takes this into account. Third, with a rising interest on targeting dietary assessments per country and populations, more indicators need to include regional differences of diet quality and environmental impacts [63]. The majority of the studies identified were conducted in developed countries which can bias the results of the review [43]. Only one study was identified in the literature review that focused on low and middle income countries [56]. When considering regional and population differences, recommendations might differ according to each context (e.g., cut-off points, reference values for specific populations) [29••, 63]. For example, a flexitarian approach to reduce the environmental impact of diets, and avoid micronutrient deficiencies instead of a full vegetarian or vegan diet, has been recommended especially for developing countries [1•], which have also showed positive health outcomes [15] and a more environmentally friendly integration of meat production into food systems [64]. In fact, even if plant-based proteins have a lower environmental impact, following a plant-protein only diet on a global scale might not be feasible and the population might not be willing to follow this dietary pattern. Thus, integrating NHE dimensions will allow for a better identification of synergies and trade-offs to define dietary recommendations that align with sustainability goals.

This paper aimed to review diet quality indices and health indices and its potential use for environmental assessment of foods, meals and diets. The review identified three main areas that need to be clearly defined when using diet quality indices in the environmental evaluation of foods. First, it has to be clearly stated which dimension of diet quality is measured and use an index accordingly. Second, methodological adaptations need to be defined in detail and justified depending on the aims of the study. For example, capping should mainly be applied when analysing whole diets and dietary reference intake or dietary reference guidelines should be adapted to the target population and potential deficiencies. Thirdly, one should discuss how the integration into the environmental assessment is performed (parallel, scaled or integrated).

## Conclusions

Traditionally, nutrition and environmental sciences have been separate disciplines. However, increased recognition of the impact of food production and consumption on climate change has identified a need for combined metrics to measure both diet quality and environmental impacts of diets. However, there remains a lack of information on methodology to integrate both disciplines. This literature review highlighted the complexity of capturing all aspects of diet quality and health impacts of foods. The results of this review identified five different categories of indices that can be potentially used for combined NHE analysis: group 1: nutrient-food quantity-based; group 2: guideline-based; group 3: diversity-based; group 4: nutrient quality-based; and group 5: health-based. When assessing the nutritional dimension, nutrient profiling indices and guidelines-based indices were identified as the most used ones, but a more holistic analysis is needed to capture all aspects of diet quality. The literature review identified less indices evaluating health, due to its complexity, but epidemiological dietary risk factors seem a promising approach to include health aspects when evaluating the environmental impact of foods. When including nutritional and health dimensions on the environmental assessment of foods, the analysis can be performed either parallel, scaled or integrated. While a more integrative approach is best able to identify synergies and trade-offs between dimensions, a parallel or scaled approach is still most commonly used. The review highlighted the following recommendations: (1) use the most appropriate diet quality-health index to answer your research question (depending on which aspect of diet quality you want to capture); (2) include as many aspects of diet quality and health on your analysis; (3) apply the index correctly and adjust methodological aspects to your dataset; (4) properly define the applied methodology of the index to ensure transparency and comparability of results; (5) when possible include an integrated approach when performing a NHE assessment of food to ensure trade-offs and synergies between dimensions are well captured. 6) Future research should focus on a more holistic sustainable assessment that can represent more dimensions of food.

### Supplementary Information

Below is the link to the electronic supplementary material.

Supplementary file1 (DOCX 46 KB)

## Data Availability

The relevant data are included in the manuscript and in the supplementary information.
