# Supplementary material for: Review of Diet Quality Indices that can be Applied to the Environmental Assessment of Foods and Diets
Source: Curr Nutr Rep. 2024 Apr 16;13(2):351–62. doi: 10.1007/s13668-024-00540-0 (PMC11133024; doi:10.1007/s13668-024-00540-0)
Supplement: Supplementary file 1 — Supplementary file1 (DOCX 46 KB) [file 13668_2024_540_MOESM1_ESM.docx]

***Table S1.*** *Studies included in the literature review classified by type of index evaluated*

| **Studies included** | **Group A:**  **Nutrient/food quantity-based indices** | | **Group B:**  **Guideline based**  **indices** | **Group C:**  **Diversity based indices** | **Group D:**  **Nutrient quality-based**  **indices** | **Group E:**  **Health based**  **indices** |
| --- | --- | --- | --- | --- | --- | --- |
|  | **A1** | **A2** |  |  |  |  |
| **Machado et al. (2023)** |  |  |  |  |  |  |
| **McAuliffe et al. (2023)** |  |  |  |  |  |  |
| **Guo et al. (2022)** |  |  |  |  |  |  |
| **Jolliet (2022)** |  |  |  |  |  |  |
| Tan et al. (2022) |  |  |  |  |  |  |
| Peonides et al. (2022) |  |  |  |  |  |  |
| van der Bend et al. (2022) |  |  |  |  |  |  |
| Hercberg et al. (2022) |  |  |  |  |  |  |
| **McLaren et al. (2021)** |  |  |  |  |  |  |
| **Stylianou et al. (2021)** |  |  |  |  |  |  |
| **Aldaya et al. (2021)** |  |  |  |  |  |  |
| **Strid et al. (2021)** |  |  |  |  |  |  |
| Verger et al. (2021) |  |  |  |  |  |  |
| Drewnowski et al. (2021) |  |  |  |  |  |  |
| **Weidema and Stylianou (2020)** |  |  |  |  |  |  |
| **Green et al. (2020)** |  |  |  |  |  |  |
| **Bianchi et al. (2020)** |  |  |  |  |  |  |
| Miller et al. (2020) |  |  |  |  |  |  |
| Hlaing-hlaing et al. (2020) |  |  |  |  |  |  |
| Drewnowski and Fulgoni (2020) |  |  |  |  |  |  |
| Trijsburg et al. (2019) |  |  |  |  |  |  |
| **Eme et al. (2019)** |  |  |  |  |  |  |
| **Berardy et al. (2019)** |  |  |  |  |  |  |
| Burggraf et al. (2018) |  |  |  |  |  |  |
| **Hallström et al. (2018)** |  |  |  |  |  |  |
| **van Dooren et al. (2017)** |  |  |  |  |  |  |
| **Saarinen et al. (2017)** |  |  |  |  |  |  |
| **Röös et al. (2015)** |  |  |  |  |  |  |
| Gil et al. (2015) |  |  |  |  |  |  |
| Fern et al. (2015) |  |  |  |  |  |  |
| WHO (2015) |  |  |  |  |  |  |
| **Van Kernebeek et al. (2014)** |  |  |  |  |  |  |
| **Heller et al. (2013)** |  |  |  |  |  |  |
| Ocké (2013) |  |  |  |  |  |  |
| Chiuve et al. (2012) |  |  |  |  |  |  |
| WHO (2010) |  |  |  |  |  |  |
| Wirt and Collins (2009) |  |  |  |  |  |  |
| Fulgoni et al. (2009) |  |  |  |  |  |  |
| Fransen and Ocké (2008) |  |  |  |  |  |  |
| Guenther et al. (2008) |  |  |  |  |  |  |
| Waijers et al. (2007) |  |  |  |  |  |  |
| Drewnowski (2005) |  |  |  |  |  |  |

***Footnote:*** *Studies in bold include the environmental dimension assessment*
